# Cross cultural adaptation and validation of Nepali Version of Activity Scale for Kids (ASK)

**DOI:** 10.1186/s41687-022-00479-2

**Published:** 2022-06-16

**Authors:** Regan Shakya, Renuka Suwal, Ishwar Adhikari, Jasmine Shrestha, Subham Gyawali, Archana Shrestha

**Affiliations:** 1 Department of Physiotherapy, Kathmandu University School of Medical Sciences, Dhulikhel Hospital, Kavre, Nepal; 2 Research Center for Rehabilitation Division, CBR Bhaktapur, Bhaktapur, Nepal; 3 Department of Community Programs, Kathmandu University School of Medical Sciences, Dhulikhel Hospital, Kavre, Nepal

**Keywords:** Self-report, Translations, Psychometrics, Outcome assessment

## Abstract

**Background:**

Activity Scale for Kids (ASK) is self reported, widely used tool to measure the physical disability in children aged 5–15 years. It has two versions; ASK-performance version and ASK-capability version, both with excellent psychometric properties in English and other translated languages. However, the tool is not available in Nepali. The aim of our study is to translate, culturally adapt and validate the tool in the context of the Nepali population.

**Methods:**

A standard translation guideline was used to translate both the versions of ASK tool into the Nepali language. One hundred and two participants were assessed to establish the reliability and validity of the tool. Internal consistency and test retest reliability was established using cronbach’s alpha and intra class correlation coefficient. Validity was established by three methods; ceiling and floor effects, group discriminations, and comparing the results of ASK with the Nepali version of KINDL.

**Results:**

The mean age of the sample participants were 12.74 years (SD 2.2). The internal consistency and test retest reliability for both the Nepali versions of ASK was significant at 0.98 and 0.94 respectively. The tool had a negligible ceiling effect (< 5%) but a moderate floor effect (ASKp-Np 7.8% and ASKc-Np 8.8%). It was able to discriminate between the mobility aid required for moving inside and outside the home environment. Moderate correlation was observed between the both the Nepali versions of ASK and the total score of KINDL (r = 0.5).

**Conclusions:**

Nepali version of ASK is reliable and valid tool to measure physical disability in the Nepali pediatric population.

## Background

Physical disability is referred to as the inability to carry out day to day physical functions and interaction with the environment. The disability may be present at birth, as a result of a deformity, physical deficiency, diseases or injuries sustained and are influenced by personal and environmental factors [1, 2]. The information regarding people with disabilities in Nepal is limited [3]. The Nepal census 2011 estimated the prevalence rate of disability in Nepal at 1.94% out of which one-third are physically disabled [4]. Another model study estimated disability prevalence of 3.1 in Nepal [5]. Both of these estimates are lower than the global estimate of prevalence at around 15% [6]. This study also found that globally 6% of the children aged less than 15 years were estimated to have moderate to severe disability. A Caribbean study revealed that limited information on physical disability is due to lack of cultural and language specific tool for assessing disability in low income countries [7].

Activity Scale for Kids (ASK) is a widely used self-reported questionnaire measuring the physical disability in children aged 5–15 years [8]. The tool is unique due to the inclusion of a child’s perspective in measuring their physical limitation and functional capacity. ASK has established excellent psychometric properties in English and other languages [9–12]. ASK has two versions; a performance version (ASKp) and a capability version (ASKc). The performance version takes into account the environment in which the child must function, measuring the activity of a child in that environment. The capability version considers the child’s perspective, taking into account the activities that the child thinks he/she could do despite their usual environment. The tool is feasible to administer and has demonstrated efficiency in detecting clinically important change signifying it’s clinical and research use [9, 13].

There are limited outcome measures available in Nepali to determine functional outcomes in the pediatric population. ASK has not- been translated or validated in Nepali. The aim of this study is to translate the ASK questionnaire to ensure that it is- a culturally appropriate and valid tool for use in Nepal.

## Methods

The study was conducted in two phases. During the first stage, the English version of the ASK questionnaire was translated and culturally adapted into Nepali (ASK-Np). Following this the validity of the translated ASK-Np was tested.

### ASK: English version

ASK is a 30-item self-administered tool intended to measure physical disability in children aged 5 to 15 years with functional limitations due to a wide range of different health conditions. ASK has two versions; ASK-performance (ASKp) measuring what the child usually “did do”, and ASK-capability (ASKc) measuring what the child “could do” in the last week. There are 7 sub domains represented within the ASK tool including personal care (3 items), dressing (4 items), locomotion (7 items), play (2 items), standing skills (5 items), transfers (5 items), and other skills (4 items). Each item is rated on a 5 point scale with zero denoting no physical activity to four denoting maximum physical activity. The total score is expressed in percentages calculated by sum of the child’s score on each item divided by the total score that the child could have achieved. The higher score indicates better physical function of a child with 100% indicating full physical function [8, 9].

### ASK: Translation and adaptation into Nepali

A standard and accepted procedure was used to translate and adapt the English version into Nepali language [14]. To avoid information bias, the profiles of forward and backward translators included people with both medical and non medical background. The forward translation was carried out by two translators; an experienced surgeon fluent in both English and Nepali (medical background) and a notarized non medical translator unknown to the concepts of the items and its purpose. A panel of researchers and translators synthesized the forward translations into a common Nepali version. The synthesized version was independently back translated by two translators. An expert committee composed of primary researchers, translators, an experienced social worker who works with children with disabilities and a specialized pediatric physiotherapist reviewed all the translations and written reports to reach a consensus on the Nepali translation of the questionnaire. The Nepali version and the written report of the expert committee were sent to the developers of ASK. Ten children [10–12 years] were randomly pretested for clarity and understanding of the Nepali version. Minor considerations both from the developers of ASK and the results of pretest were adjusted to produce the final Nepali version of ASK scale; ASKp-Np (ASKp-Nepali) and ASKc-Np (ASKc- Nepali).

## Validation of Nepali version of ASK

### Study setting and participants

A purposive sample of 102 children aged between 8 and 15 years with a diagnosed musculoskeletal or neuromuscular condition was recruited. Data were collected from three centers with their approval; Special Children and Research Center, CBR Bhaktapur, and Dhulikhel Hospital. Children were excluded: (a) if they were unable to read and understand the Nepali language, (b) had cognitive deficits that prevented them from understanding the tool.

### Data collection

At each center, the study details were explained to the eligible children, their parents, health professionals involved, and the social workers. Parental consent was gained prior to administering the tools. The parent was interviewed for the demographic and medical information of their child such as age (in years), gender (male/female), No. of siblings, type of education (special/ normal) and disease condition (musculoskeletal/ neuromuscular). The Nepali version of the ASK and KINDL questionnaire was administered to the children. To assess the test–retest reliability, ASK-Np was re-administered to the 55 participants who responded after the period of 14 days [15].

### KINDL

KINDL is a self administered tool to assess Health-Related Quality of Life in children aged 3 years or above [16]. It is easy to administer, with good psychometric properties and is flexible to use. It consists of 24 items divided into six domains: physical well-being (PWD), emotional well-being (EWD), self-esteem (SE), family (FM), friends (FR) and everyday functioning (school or nursery school/kindergarten) (SC). The Nepali version of KINDL was used in this study [17].

### Statistical analysis

The sample characteristics were summarized using mean (standard deviation) [range] for the continuous variables and frequency (percentage) for the categorical variables. We used Kologomorov–Smirnov’s (K–S test) for normality to determine the distribution of total scores of ASK-Np and KINDL. The significant K–S test for normality would indicate the distribution is not normal and could be skewed or normal distribution. The internal consistency was assessed using Cronbach’s alpha for total scores of ASK-Np. Similarly, test–retest reliability was assessed using Interclass Correlation Coefficient (ICC) with two way mixed model absolute agreement method for the total scores of ASK-Np. To determine the validity of the tool, (a) floor and ceiling effects of the tool were measured, (b) Mann–Whitney U test was used to compare mean scores between the groups (mobility support needed inside home environment, mobility support needed outside home environment and medical conditions) and (c) a correlation test was used to compare between the total scores of both the versions ASK-Np and the different dimensions and the total scores of KINDL [18]. We used SPSS version 21 for the statistical analysis.

## Results

### Sample description

**Fig. 1:**
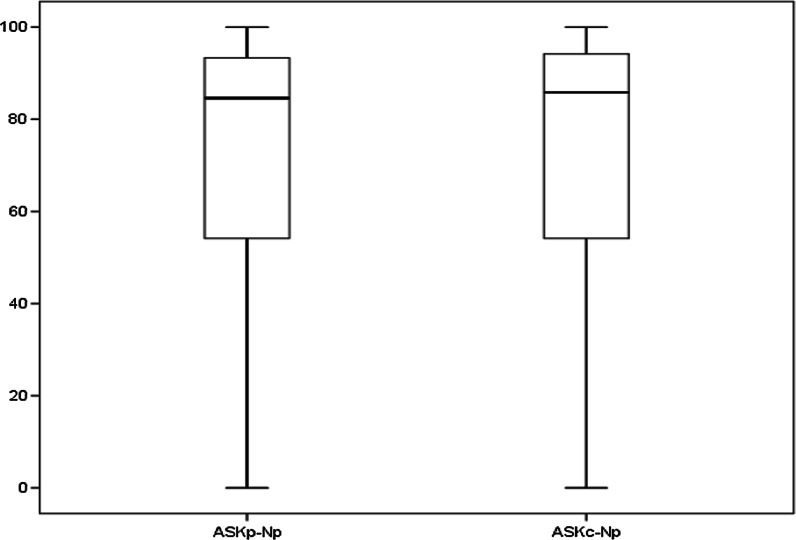
Distribution of the ASK-Np scores

The mean age of the participants was 12.74 (SD = 2.2) years. More than half were male and four- fifth of the participants had at least one sibling. The majority of the participants’ had neuromuscular disorders and was receiving special education. Forty nine percent of the participants attempted questions by themselves and three-tenths (31%) of the participants required most assistance. With respect to mobility, three- quarters of the participants did not require supporting devices while moving inside or outside of the home environment (Table 1). The distribution of total scores of ASKp-Np and ASKc-Np are shown in Fig. 1.

**Table 1:** Characteristics of study participants (n = 102)

Characteristics	Frequency (%)
Age (years), mean (SD) [range]	12.74 (2.2) [8–15]
Gender	
Male	58 (56.9%)
Female	44 (43.1%)
No. of siblings	
No siblings	18 (17.6%)
One sibling	53 (52.0%)
Two	23 (22.5%)
Three	4 (3.9%)
Four	1 (1.0%)
Five	3 (2.9%)
Type of education	
Special education	98 (96.1%)
Normal education	4 (3.9%)
Disease condition	
Musculoskeletal	7 (6.9%)
Neuromuscular	95 (93.1%)
Form completion	
All by the patient	50 (49.0%)
Questionnaire read to patient	17 (16.7%)
Some of the question	4 (3.9%)
Most of the question	31 (30.4%)
Support while moving inside home	
No	79 (77.5%)
Yes	23 (22.5%)
Support while moving outside home	
No	81 (79.4%)
Yes	21 (20.6%)
Total score ASKp-Np, mean, (SD) [range]	68.5 (34.0) [0–100]
Total score ASKc-Np, mean, (SD) [range]	69.18 (34.3) [0–100]

### Reliability

The internal consistency and Test retest reliability was excellent for the Nepali version of ASK (ASKp-Np and ASKc-Np). The internal consistency for ASKp-Np and ASKc-Np is 0.98. Similarly, the intra-class correlation coefficient is statistically significant at 0.93 for ASKp-Np and similarly the intra-class correlation coefficient is 0.92for ASKc-Np (Table 2).

**Table 2:** Internal consistency and Intra-class correlation coefficient of ASK-Np

Scale	Internal consistency (Cronbach’s alpha)	Test retest reliability (ICC)	Lower bound	Upper bound	*p*
ASKp	**0.98**	**0.93**	0.84	0.96	< 0.001
ASKc	**0.98**	**0.92**	0.83	0.96	< 0.001

The statistically significant results are in bold

### Construct validity

The ceiling effects were observed at 4.9% in both versions of ASK-Np, whereas 7.8% and 8.8% was observed at floor in the ASKp-Np and ASKc-Np respectively. When comparing groups, the participants who gained the higher total score of ASK-Np didn’t require any form of support while moving inside or outside the home environment. There was no significant relation with the different health conditions (Table 3). The distribution of the total score of KINDL was normally distributed in contrast to the distribution of the total score of both versions of ASK-Np. Therefore, we used Spearman’s rank correlation to compare the different dimensions of KINDL with the total scores of ASK-Np. We observed a mild to moderate positive correlation between the total scores of ASK with all the domains of KINDL except for the family dimension. Similarly, there was moderate correlation between the total scores of ASK-Np with the total scores of KINDL (Table 4).

**Table 3:** Group comparison of ASK-Np

	Description		n	Mean (SD)	p*
ASKp	Inside environment	No support	79	**62.35**	< 0.001
		With support	23	**14.24**	
	Outside environment	No support	81	**61.94**	< 0.001
		With support	21	**11.24**	
	Health condition	Musculoskeletal	7	61.07	0.38
		Neuromuscular	95	50.79	
ASKc	Inside support	No support	79	**62.37**	< 0.001
		With support	23	**14.15**	
	Outside support	No support	81	**61.93**	< 0.001
		With support	21	**11.26**	
	Health condition	Musculoskeletal	7	59.36	0.47
		Neuromuscular	95	50.92	

The statistically significant results are in bold

^*^Mann–Whitney U test

**Table 4:** Correlation between ASK-Np and KINDL-Np

KINDL		PWB	EWB	SE	FM	FR	SC	TT
ASKp	r	**0.24**	**0.24**	**0.48**	0.17	**0.38**	**0.22**	**0.51**
	p	0.02	0.02	< 0.001	0.09	< 0.001	0.03	< 0.001
ASKc	r	**0.20**	**0.22**	**0.48**	0.15	**0.35**	**0.21**	**0.48**
	p	0.05	0.04	< 0.001	0.13	< 0.001	0.04	< 0.001

*PWB* physical well being, *EWB* emotional well being, *SE* self esteem, *FM* family, *FR* friends, *SC* school, *TT* KINDL total score

Statistically significant results are in bold (*p* < 0.05)

## Discussion

The ASK questionnaire was translated, culturally adapted and validated into the Nepali language.

A number of items in the tool were culturally adapted with consensus from the expert committee and the developers of ASK. Items with printing or script writing and examples of dribbling a basketball are not common in Nepal; instead they were replaced with school related work and playing with a ball. The finalized Nepali version had a suffix “Np” added to the tool acronym to identify it as the Nepali version of ASK: ASK-Np (ASK-Nepali); subsequently ASKp-Np (performance version in Nepali), ASKc-Np (capability version in Nepali). Good psychometric properties were obtained for the tool. The reliability was determined by internal consistency and test retest analysis. The internal consistency of 0.98 for both the ASK-Np versions is above the recommended value [19]. Similarly, the test retest analysis established an excellent agreement for both the versions. The findings are consistent with the original tool and with the other translated versions [9, 10, 20].

The validity of ASK-Np was tested using three methods: a) the assessment of ceiling and floor effects, b) score discrimination among different groups, and c) comparison between the total scores of ASK-Np with KINDL-Np and its dimensions.

The ceiling and floor effect indicates the ability of a tool to distinguish between responses at the extreme ends of the tool [18]. In this study, it was found that both the versions of ASK-Np demonstrated negligible ceiling effects (< 5%). The floor effect was observed to be moderate for ASKp-Np (7.8%) and ASKc-Np (8.8%). The results of this study partially agree with the original tool which reported the negligible ceiling effect of 4% and with zero floor effect [9]. The differences in the floor effect in this study could be attributed to the severity of the participants’ neuromuscular conditions which included limited physical functioning capabilities [21]. A UK based study reported some participants scored zero on the scores of ASK. They also determined that the impaired functional mobility as a significant factor affecting the scores of ASK [22].

Construct validity was determined by the known-groups validity, which is assessed by comparing the target outcomes scores of different pre-specified groups that are known to vary on the construct of interest. The participant’s physical functioning outcomes in different environments (inside and outside) and use of mobility aids (with support/ no support) are compared. It is hypothesized that the individual who requires the support of mobility aids would have lower physical functioning capabilities [21–23]. Significant difference was observed in the scores of both the versions of ASK-Np when compared with use of mobility aid during physical functioning in different environments. The group which required support for mobility produced significantly lower scores in both the versions (ASKp-Np and ASKc-Np) with inside and outside environments. In contrast the group which didn’t require support for mobility produced significantly higher scores in ASK-Np. The findings of this study are consistent with the findings observed in the Portuguese version [10]. In comparison to the Portuguese study, we didn’t observe significant difference between the scores of ASK-Np when comparing the groups with different health condition. The finding suggests similar level of physical functioning in different health condition in contrast to the findings of the Portuguese version [10]. The sample for musculoskeletal conditions in Nepali version was significantly lower therefore it is difficult to draw conclusions on physical functioning in different health conditions.

In the next validation method, the scores of ASK-Np were compared with the scores of KINDL. Multiple studies have demonstrated direct positive relation between lower limb physical function with quality of life scores [24, 25]. Similar strategies of validation were used in original tool and the other translated versions. Moderate correlation (r = 0.5) was observed between the total scores of both versions of ASK-Np and the total score of KINDL corresponding to the findings with the previous studies [9, 10]. Similarly, we observed mild correlation between the total scores of both the version of ASK-Np with the dimensions of KINDL except for the family dimension. The weaker correlations were observed with dimensions of physical well being, emotional well being and school. Self esteem and friends dimension demonstrated mild to moderate correlation [26]. Similar findings were observed in the Portuguese version [10].

### Limitations of the study

This study had a few limitations; moderate floor effects were observed which could be due to the limited sample size and distribution. Secondly, the majority of the sample had neuromuscular conditions; therefore conclusions could not be drawn for the children with musculoskeletal conditions. Thirdly, due to limited performance related outcome measures available in Nepali, we were not able to state the correlation with other related outcome measures for validity. We recommend the further studies to use the quantitative functional outcomes to strengthen the validity of the scale.

## Conclusion

The two versions of ASK-Np are valid and reliable tools for clinical use in Nepal to measure physical disability. The questionnaire has been culturally adapted for use by Nepali children in reporting limitations in physical functions.

## Data Availability

The dataset generated and/or analyzed during the current study are available from the corresponding author on a reasonable request.

## Abbreviations

ASK: Activities Scale for Kids
ASKc: Activities Scale for Kids capability version
ASKp: Activities Scale for Kids performance version
ASK-Np: Activities Scale for Kids-Nepali version
ASKc-Np: Activities Scale for Kids-Nepali capability version
ASKp-Np: Activities Scale for Kids-Nepali performance version
CP: Cerebral palsy
EWB: Emotional well being
FM: Family
FR: Friends
KS test: Kolgomorov–Smirnov’s test
PWB: Physical well being
SD: Standard deviation
SPSS: Statistical Package for the Social Sciences
SE: Self-esteem
SC: School

## References

[1] World Health Organization (2021) Disability and health [Online]. Cited 12th Jan 2022. https://www.who.int/news-room/fact-sheets/detail/disability-and-health

[2] Centers for Disease Control and Prevention (2020) Disability & Health overview: impairments, activity limitations, and participation restrictions [Online]. Cited 12th Jan 2022. https://www.cdc.gov/ncbddd/disabilityandhealth/disability.html

[3] Rohwerder B (2020) Nepal situational analysis. Disability inclusive development. Institute of Development Studies [Report]. Cited 12th Jan 2022. https://opendocs.ids.ac.uk/opendocs/handle/20.500.12413/15510

[4] Government of Nepal (2012) National population and housing census 2011 [National report]. Cited 12th Jan 2022. https://unstats.un.org/unsD/demographic/sources/census/wphc/Nepal/Nepal-Census-2011-Vol1.pdf

[5] Eide AH, Neupane S, Hem KG (2016) Living conditions among people with disability in Nepal [Report]. Report no: SINTEF A27656

[6] World Health Organization (2011) World report on disability [online]. Cited 12th Jan 2022. https://www.who.int/publications/i/item/9789241564182

[7] Schmid K, Vézina S, Ebbeson L (2008) Disability in the Caribbean. A study of four countries: a socio-demographic analysis of the disabled. UNECLAC Statistics and Social Development Unit. Cited 12th Jan 2022. https://repositorio.cepal.org/handle/11362/5059

[8] Young NL, Yoshida KK, Williams JI, Bombardier C, Wright JG (1995). The role of children in reporting their physical disability. Arch Phys Med Rehabil.

[9] Young NL, Williams JI, Yoshida KK, Wright JG (2000). Measurement properties of the activities scale for kids. J Clin Epidemiol.

[10] Paixão D, Cavalheiro LM, Gonçalves RS, Ferreira PL (2016). Portuguese cultural adaptation and validation of the Activities Scale for Kids (ASK). Jornal de pediatria.

[11] Lawitschka A, Brunmair M, Bauer D, Zubarovskaya N, Felder-Puig R, Strahm B (2021). Psychometric properties of the Activities Scale for Kids-performance after allogeneic hematopoietic stem cell transplantation in adolescents and children: results of a prospective study on behalf of the German-Austrian-Swiss GVHD Consortium. Wien Klin Wochenschr.

[12] Fabbri L, Neviani R, Festini F, Costi S (2016). Transcultural validation of Activities Scale for Kids (ASK): translation and pilot test. Acta Biomed.

[13] Plint AC, Gaboury I, Owen J, Young NL (2003). Activities scale for kids: an analysis of normals. J Pediatr Orthop.

[14] Beaton DE, Bombardier C, Guillemin F, Ferraz MB (2000). Guidelines for the process of cross-cultural adaptation of self-report measures. Spine.

[15] Carmines EG, Zeller RA (1994). Reliability and validity assessment.

[16] Ravens-Sieberer U, Bullinger M (1998). Assessing health-related quality of life in chronically ill children with the German KINDL: first psychometric and content analytical results. Qual Life Res.

[17] Yamaguchi N, Poudel KC, Poudel-Tandukar K, Shakya D, Ravens-Sieberer U, Jimba M (2010). Reliability and validity of a Nepalese version of the Kiddo-KINDL in adolescents. Biosci Trends.

[18] Terwee CB, Bot SD, de Boer MR, van der Windt DA, Knol DL, Dekker J, Bouter LM, de Vet HC (2007). Quality criteria were proposed for measurement properties of health status questionnaires. J Clin Epidemiol.

[19] Bland JM, Altman DG (1997). Cronbach’s alpha. BMJ.

[20] Young NL, Yoshida KK, Williams JI, Bombardier C, Wright JG (1995). The role of children in reporting their physical disability. Arch Phys Med Rehabil.

[21] Orlin MN, Palisano RJ, Chiarello LA, Kang LJ, Polansky M, Almasri N, Maggs J (2010). Participation in home, extracurricular, and community activities among children and young people with cerebral palsy. Dev Med Child Neurol.

[22] Morris C, Kurinczuk JJ, Fitzpatrick R, Rosenbaum PL (2006). Do the abilities of children with cerebral palsy explain their activities and participation?. Dev Med Child Neurol.

[23] Keawutan P, Bell K, Davies PS, Boyd RN (2014). Systematic review of the relationship between habitual physical activity and motor capacity in children with cerebral palsy. Res Dev Disabil.

[24] Hall SA, Chiu GR, Williams RE, Clark RV, Araujo AB (2011). Physical function and health-related quality-of-life in a population-based sample. Aging Male.

[25] Bindawas SM, Al Snih S, Ottenbacher AJ, Graham J, Protas EE, Markides KS, Ottenbacher KJ (2015). Association between lower extremity performance and health-related quality of life in elderly Mexican Americans. J Aging Health.

[26] Akoglu H (2018). User's guide to correlation coefficients. Turk J Emerg Med.

